# SP1-Mediated Upregulation of circFAM126A Promotes Proliferation and Epithelial-Mesenchymal Transition of Oral Squamous Cell Carcinoma *via* Regulation of RAB41

**DOI:** 10.3389/fonc.2022.715534

**Published:** 2022-02-14

**Authors:** Jun Wang, Shaobo Ouyang, Siyu Zhao, Xianhua Zhang, Mingyang Cheng, Xin Fan, Ying Cai, Lan Liao

**Affiliations:** ^1^ Oral and Maxillofacial Surgery, Second Affiliated Hospital of Nanchang University, Nanchang, China; ^2^ Department of Oral Prosthodontics, Affiliated Stomatological Hospital of Nanchang University, Jiangxi Provinial Key Laboratory of Oral Biomedicine, Nanchang, China

**Keywords:** oral squamous cell carcinoma, circFAM126A, miR-186, RAB41, proliferation, epithelial-mesenchymal transition

## Abstract

**Background:**

Accumulating evidence indicates that circular RNAs have major roles in the progression of human cancers. Nevertheless, the molecular mechanism and effects of circFAM126A in oral squamous cell carcinoma (OSCC) remain unclear.

**Methods:**

Quantitative real-time PCR (qRT-PCR) was used to detect expression levels of circFAM126A in OSCC tumor tissues and cell lines; the effects of circFAM126A small hairpin RNA (shRNA) on the proliferation, migration, and invasion of OSCC cells were detected by MTT, colony formation, and transwell assays; xenograft mouse models were used to determine the effects of circFAM126A shRNA on the growth of OSCC tumors *in vivo*; the expression of miR-186 and RAB41 in OSCC tissues and cells was examined by qRT-PCR; the targeting relationship between circFAM126A and miR-186 was verified by dual-luciferase reporter and RNA pull-down assays; and the relationship between miR-186 and RAB41 was explored.

**Results:**

The expression of circFAM126A was significantly upregulated in OSCC tissues and cells. The transcription factor SP1 transcriptionally activated circFAM126A. However, knockdown of circFAM126A markedly suppressed the proliferation, migration, and invasion of OSCC cells *in vitro* and inhibited tumor growth and distant metastasis *in vivo*. Moreover, circFAM126A increased the expression of RAB41 and promoted its mRNA stability *via* binding to miR-186 and RNA-binding protein FUS. Overexpression of RAB41 antagonized the effects of circFAM126A knockdown and induced an aggressive phenotype of OSCC cells.

**Conclusion:**

SP1 transcriptionally activated circFAM126A modulated the growth, epithelial-mesenchymal transition (EMT) of OSCC cells *via* targeting the miR-186/FUS/RAB41 axis, suggesting that circFAM126A is a potential biomarker for the treatment of OSCC.

## Introduction

Oral squamous cell carcinoma (OSCC) is the most common oral malignant tumor worldwide (1). Although strategies for the diagnosis and treatment of OSCC have improved significantly in recent years, the 5-year overall survival rate for patients with advanced OSCC remains poor (2, 3). Therefore, further exploration of the pathogenesis of OSCC and identification of novel therapeutic targets, as well as potential diagnostic and prognostic biomarkers, may provide new opportunities for early diagnosis and treatment of OSCC.

Accumulating evidence has unveiled the roles of circular RNAs (circRNAs; a type of non-coding RNA) in cancers including bladder cancer (4), colorectal cancer (5), and cervical cancer (6). Abnormally expressed circRNAs can be used as diagnostic markers and targets for therapeutic intervention for various malignant tumors (7, 8). In OSCC, circRNAs can function as tumor suppressors or oncogenes (9). For example, hsa-circ-0008035, hsa-circ-0000670, and hsa-circ-0003159 have been found to be associated with OSCC tumorigenesis (10–12). However, the roles of circ_0001682 (circFAM126A), which is located on chromosome 7 with a spliced length of 181 base pairs, have not been elucidated.

Functionally, circRNAs function as competing endogenous RNAs (ceRNAs) and sponge microRNAs (miRNAs) to regulate gene expression and numerous biological processes, including proliferation, apoptosis, migration, and invasion of cancer cells (13–15). miRNAs pair with mRNA bases of target genes to induce silencing complex RISC, which further degrades the mRNA or inhibits its translation. The circRNA/miRNA/mRNA axis has been verified to be a regulator of multiple tumor-related pathways and to modulate tumorigenesis. For example, aberrant expressed circAKT1 induces malignant behaviors of cervical cancer cells *via* regulating the miR-942-5p/AKT1 axis (6); the circ-0067934/miR1324/FZD5 axis promotes the progression of hepatocellular carcinoma (16); and has-circ-0000670 promotes the proliferation, migration, and invasion of OSCC *via* regulating miR384/SIX4 axis (11).

Epithelial-mesenchymal transition (EMT) is considered as a classical theory for tumor metastasis (17). The processes of EMT is accompanied by the loss of epithelial function and acquisition of mesenchymal characteristics, which loses cell adhesion and enhances migration and invasion ability (18). Presently, increasing evidence has revealed the potentials of circRNAs in the EMT processes (19). For instance, circ_0008305 suppresses the EMT and metastasis of non-small cell lung cancer *via* miR-429/miR-200b-3p/TIF1γ axis (20). circPRRC2A-induced upregulation of TRPM3 promotes the EMT, angiogenesis and metastasis of renal cell carcinoma (21). circIGHG enhances the EMT of OSCC *via* regulating miR-142-5p/IGF2BP3 axis (22). However, the study on the roles of circRNAs in OSCC is still limited.

In this research, we investigated the roles of circFAM126A in OSCC tissues and cell lines using circRNA microarrays, bioinformatics, and functional studies. We found that circFAM126A could function as an oncogene in OSCC, and that its knockdown suppressed the proliferation and EMT of OSCC cells *via* regulation of the miR-186/RAB41 axis.

## Materials And Methods

### Tissue Samples and Cell Lines

A total of 30 OSCC patients who underwent surgeries at the Affiliated Stomatological Hospital of Nanchang University were involved in this research. Adjacent normal tissues were taken 5 cm away from the edge of the tumor. The diagnosis of OSCC was confirmed by histological examination. Patients with OSCC who had received prior treatment for their tumor or had a history of other solid tumors were excluded. This study was approved by the Human Research Ethics Committee of the Affiliated Stomatological Hospital of Nanchang University. Informed consent was obtained from all patients.

OSCC cell lines CAL27, SCC25, SCC15, TSCCA, UM1 and UM2, normal human oral epithelial cells (NHOK), and HEK-293 T cells were obtained from the Institute of Biochemistry and Cell Biology of the Chinese Academy of Sciences (Shanghai, China). All cell lines were cultured in 90% RPMI-1640 medium (Gibco, USA) with 10% fetal bovine serum (FBS) and 1% penicillin–streptomycin solution (Invitrogen) at 37°C in a moist atmosphere with 5% CO_2_.

### Microarray Analysis

The microarray data set GSE131182 analysis was performed with Limma R Human CBC circRNA under the following the standard: |logFC|> 2 and P<0.05. The number of differentially expressed circRNAs was 417, among which 383 circRNAs were upregulated and 34 downregulated.

### RNase R Treatment

To prove that circFAM126A is a circRNA, total RNA from CAL27 and UM1 was treated with RNase R (Sigma) at 37°C for 15 min and then purified with phenol-chloroform (Sigma). The expression of circular or linear FAM126A was determined by quantitative real-time polymerase chain reaction (qRT-PCR).

### Actinomycin D

The circFAM126A plasmids were transiently transfected into OSCC cells using Lipofectamine 2000 (Invitrogen, Carlsbad, CA, USA). After 24 h, actinomycin D (5µg/mL) was added to the culture medium, followed by incubation for 0 h, 4 h, or 8 h, 16 h, and 24 h. mRNA stability was analyzed by PCR.

### qRT-PCR

TRIzol™ reagent (Invitrogen, CA, USA) was used to extract total RNA according to the manufacturer’s instructions. To ensure the purity of circRNAs, RNase R (Geneseed, Guangzhou, China) was used to digested the RNAs for 20 min. The RNA concentration was measured using a Nanodrop 2000 (Thermo, USA), and qRT-PCR was performed on a 7500 Fast Real-Time PCR System (Applied Biosystems, Thermo Fisher Scientific) using a SYBR Premix Ex Taq II kit (Takara Bio, Beijing, China) to examine the relative expression of circRNAs. GAPDH was used to normalize circRNA expression levels.

First, total RNA was reverse transcribed into cDNAs by TaqMan Reverse-transcription. The cDNAs were synthesized using a PrimeScript RT reagent kit (Takara, Tokyo, Japan) transcriptase, random 6mers, RNase inhibitor, Oligo dT primer, dNTP mixture, and reaction buffer. The cycle conditions were 95°C for 30 s (initial denaturation), followed by 95°C for 5 s and 60°C for 34 s, for 40 cycles. We used the 2^−ΔΔCt^ method to analyze the data (23).

### Cell Transfection

The circRNA small hairpin RNA (shRNA), miR-186 mimics and inhibitor, and specific negative control were synthesized and purchased from GenePharm (Shanghai, China). The circFAM126A vectors were constructed with amplified DNA fragments, including the sequence of exons 15 and 16 of the PTK2a gene with flanking introns containing complementary Alu elements (GeneChem, Shanghai, China). Cells were transfected using Lipofectamine 3000 (Invitrogen, CA, USA) for 48 h.

### Cell Viability (MTT) Assay

Logarithmic-phase cells were digested with trypsin, collected, and used to prepare a cell suspension after centrifugation. A mixture of the cell suspension containing 100 μl cells was added to each well of a 96-well-plate. The cells were cultured in an incubator at 37°C with 5% CO_2_. Cells were supplemented with 10 μl MTT solution (5 mg/ml, 0.5% MTT) and further cultured for 4 h. Then, 150 μl dimethyl sulfoxide was added to each well, followed by shaking at low speed for 10 min to fully dissolve the crystals. The absorbance values of each well were measured using a microplate reader at an optical density of 490 nm.

### Colony Formation Assay

After 48 h transfection, cells were digested with trypsin. A cell suspension was prepared in complete medium. Then cells were washed with phosphate-buffered saline. Trypsin was added and the cells were centrifuged. The cells were seeded into six-well plates (500–1000 cells/well), shaken gently, and cultured for 14 days. Then, 1000 μl impurity-free crystal violet dye was added to the cells. Cells were visualized under a microscope.

### Transwell Cell Migration and Invasion Assays

Transwell assays were used to determine the invasion and migration ability of the OSCC cells. After transfection for 48 h, cell culture transwell inserts (8-mm pore size; Falcon; BD Biosciences) were placed in 48-well plates in the upper chamber with or without precoated Matrigel (BD Biosciences, San Jose, CA, USA). The membrane was hydrated with FBS 2 h prior. Cells in the lower chamber were cultured with RPMI-1640 (600 µl) containing 10% FBS. After 24 h, the migrated or invaded cells were fixed with 100% methanol and cultured with crystal violet. The numbers of migrated and invaded cells were counted under a microscope.

### RNA Probe Pull-Down Assay

Biotinylated probes binding to the junction region of circFAM126A or miR-186 were designed by GenePharm. The oligonucleotide probe was used as a negative control. Approximately 1×10^7^ cells were lysed in lysis buffer and incubated with 3 μg biotinylated probe for 2 h. Cell lysates were incubated with streptavidin magnetic beads (Life Technologies, Gaithersburg, MD, USA) for 4 h to pull down the biotin-conjugated RNA complex. Cells were washed with lysis buffer five times. Subsequently, the bound miRNA in the pull-down complex was extracted using TRIzol reagent and analyzed by qRT-PCR.

### Wound Healing Assay

Cells were seeded into 6-well plates. Then cells, cells at 80% confluence, were scratched using 20 μl pipette tip. Afterwards, cells were cultured with DMEM medium for 0 h and 24 h and captured using an inverted microscope.

### Dual-Luciferase Reporter Assay

Target analyses of circFAM126A and miR-186 (https://starbase.sysu.edu.cn/index.php), and of miR-186 and RAB41 (http://www.targetscan.org/vert_72/), were performed on the biological prediction, respectively. The sequences of the circFAM126A and RAB1 3′ untranslated regions (UTRs) containing the miR-186 binding sites were cloned into luciferase reporter vectors (Promega, Madison, WI, USA) to form the wild-type vectors wt-circFAM126A and wt-RAB41. The mutant luciferase reporter vector constructs mut-circFAM126A 3’UTR and mut-RAB41 were created by mutating the binding sites of miR-186. For the dual-luciferase reporter assay, HEK-293 T cells were co-transfected with these vectors and an miR-186 mimic or negative control. After 48 h, luciferase activity was measured by using a dual-luciferase reporter assay system (Promega).

### Chromatin Immunoprecipitation (ChIP) Assay

ChIP assay was performed using a ChIP Kit (Millipore, USA). Briefly, cells were fixed in 1% formaldehyde. Afterwards, crosslinked chromatin was ultra-sonicated, and immunopreciated with anti-SP1 or control anti-IgG bound-protein G beads. The enrichments of DNA fragments were determined using qRT-PCR.

### Mouse Xenograft Tumor Models

Mouse xenograft tumor models were established for *in vivo* assays. OSCC cells transfected with circFAM126A shRNA were subcutaneously injected into nude mice. The volumes of xenograft tumors were measured every week for 6 weeks. At the end of week 6, mice were sacrificed, and weights of tumors were measured. Immunohistochemical staining was performed to determine the expression of Ki67. This animal study was approved by the Animal Care Board of the Affiliated Stomatological Hospital of Nanchang University.

### Statistical Analysis

Each experiment was conducted three times. Statistical analysis was performed with SPSS v.22.0 (IBM, SPSS, Chicago, IL, USA). Data were presented as mean ± standard deviation. Comparisons between two groups were performed using student’s t-test. Differences among multiple groups were evaluated with one-way analysis of variance. A *P*-value less than 0.05 was considered to indicate statistical significance.

## Results

### circFAM126A Is Overexpressed in OSCC Tissues and Cells

**Figure 1:**
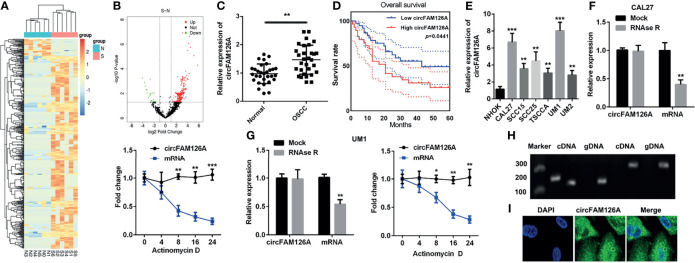
circFAM126A is upregulated in OSCC. **(A, B)** Microarray analysis the differentially expressed circRNAs in OSCC. **(C)** Expression of circFAM126A in clinical samples. **(D)** The overall survival of OSCC patients. **(E)** Expression of circFAM126A in OSCC cells. **(F)** Relative RNA levels and RNA stability in CAL27 detected by qRT-PCR. **(G)** Relative RNA levels and RNA stability in UM1 detected by qRT-PCR. **(H)** Primers amplified in cDNA or gDNA determined by qRT-PCR. **(I)** Locations of circFAM126A detected by fluorescence assay. S: OSCC patients; N: normal group. *P<0.05, **P<0.01, ***P<0.001.

**Figures 1A, B** showed the differentially expressed circRNAs in OSCC patients (S) and healthy control (N), among which 383 circRNAs were upregulated and 34 downregulated. The expression of circFAM126A was more remarkable. We further determined the expression of circFAM126A in OSCC tissues and cells using qRT-PCR. As shown in **Figure 1C**, the expression of circFAM126A was significantly higher in OSCC tissues compared with healthy control (*P*<0.01). Moreover, high expression of circFAM126A was significantly associated with gender, tumor stage, and lymph node metastasis, but not with age (**Table 1**); additionally, high level of circFAM126A was associated with poor overall survival (**Figure 1D**, *P*<0.05). The expression of circFAM126A was significantly increased in OSCC cells, such as CAL27, SCC15, SCC25, TSCCA, UM1 and UM2 (**Figure 1E**, *P*<0.01, *P*<0.001), which was more remarkable in CAL27 and UM1 cells. Therefore, CAL27 and UM1 cells were used in the following experiments. To further verify the circRNA characteristics of circFAM126A, RNAse and PCR assays were performed. As shown in **Figures 1F, G**, circFAM126A was stable and could resist RNase R digestion in CAL27 and UM1 cells (*P*<0.05, *P*<0.01). Moreover, circFAM126A could be amplified in cDNA, but not gDNA (**Figure 1H**). Furthermore, fluorescence imaging showed that circFAM126A was located in the nucleus as well as in the cytoplasm (**Figure 1I**).

**Table 1:** Clinical features of OSCC patients.

Parameters	*Total*	*circFAM126A Expression*		*P* value
		High	Low	
*Tissues*				<0.01
* OSCC*	30	21	9	
* Health*	30	7	23	
*Age*				0.0157
* ≥60*	31	19	12	
* <60*	29	15	14	
*Gender*				0.0363
* Male*	33	23	10	
* Female*	27	11	16	
*Stage*				0.6452
* I-II*	6	2	4	
* III-IV*	24	21	3	
*Lymph node metastasis*				<0.01
	30	22	8	

### SP1 Transcriptionally Activates circFAM126A in OSCC

**Figure 2:**
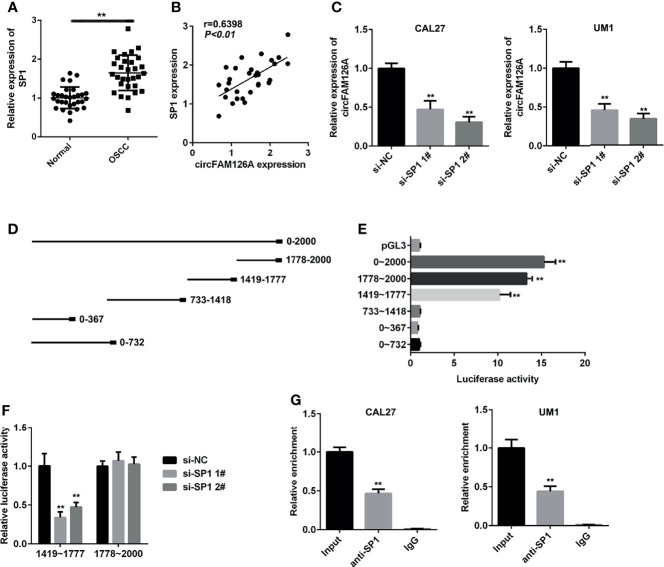
SP1 transcriptionally activates circFAM126A in OSCC. **(A)** mRNA expression of SP1 in OSCC patients measured using qRT-PCR. **(B)** Correlation analysis of expression levels of SP1 and circFAM126A in OSCC patients. **(C)** Expression of circFAM126A detected by qRT-PCR. **(D)** Serial truncations of circFAM126A promoter inserted into pGL3 vector. **(E, F)** Interaction between SP1 and circFAM126A verified by luciferase assay. **(G)** Interaction between SP1 and circFAM126A verified by ChIP assay. **P<0.01.

SP1 is a crucial transcription factor and functions as an oncogene in OSCC. As shown in **Figure 2A**, the expression of SP1 was significantly higher in patients OSCC tissues compared with healthy control (*P*<0.01). The expression of SP1 in OSCC samples was positively correlated with circFAM126A (**Figure 2B**, *P*<0.01). Moreover, knockdown of SP1 significantly decreased expression levels of circFAM126A (**Figure 2C**, *P*<0.01). To further verify the interaction between SP1 and circFAM126A, serial truncations of the circFAM126A promoter were inserted into the pGL3 vector in HEK-293 T cells. The luciferase activity was significantly increased when the 1419–1477 and 1778–2000 truncations were used (**Figures 2D, E**, *P*<0.01). Furthermore, knockdown of SP1 significantly suppressed luciferase activity in the 1419–1477 group (**Figure 2F**, *P*<0.01). The ChIP further verified that SP1 bound to the promoter of circFAM126A (**Figure 2G**, *P*<0.01).

### Knockdown of circFAM126A Inhibits the Proliferation and EMT of OSCC Cells *In Vitro*

**Figure 3:**
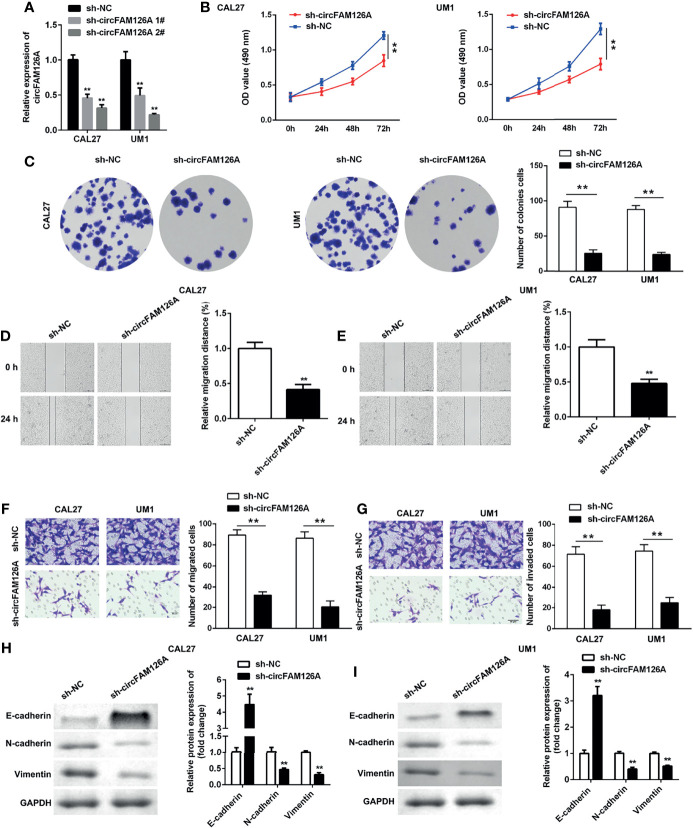
circFAM126A shRNA inhibits proliferation, migration, and invasion of OSCC cells. **(A)** Expression of circFAM126A in OSCC cells detected by qRT-PCR. **(B)** Viability of CAL27 and UM1 cells determined by MTT assay. **(C)** Proliferation of OSCC cells detected by colony formation assay. **(D, E)** Migration detected using wound healing assay. **(F, G)** Migration and invasion of OSCC analyzed by transwell assay. **(H, I)** Protein expression determined using western blot. **P<0.01.

Next, to further explore the roles of circFAM126A in OSCC, we treated CAL27 and UM1 cells with circFAM126A shRNA. This knockdown of circFAM126A effectively reduced the expression levels of circFAM126A, with a particularly marked effect in the sh-circFAM126A 2# group (**Figure 3A**, *P*<0.01). Therefore, sh-circFAM126A 2# was used in the following experiment. Knockdown of circFAM126A also significantly inhibited the viability and proliferation of OSCC cells (**Figures 3B, C**, *P*<0.01), and markedly inhibited the migration and invasion of CAL27 and UM1 cells *in vitro* (**Figures 3D–G**, *P*<0.01). Moreover, circFAM126A knockdown significantly suppressed the protein expression of Snail, Vimentin, and N-cadherin, but increased E-cadherin (**Figures 3H, I**, *P*<0.01).

### Knockdown of circFAM126A Inhibits Growth of OSCC Tumor *In Vivo*

**Figure 4:**
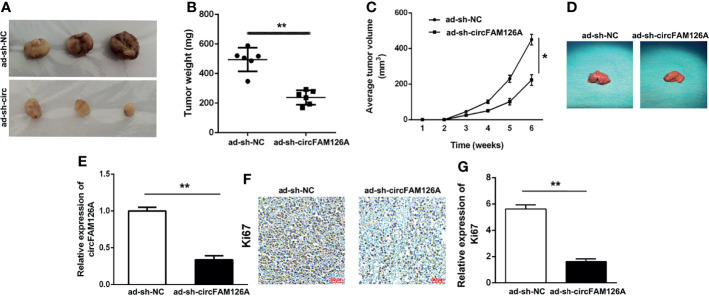
circFAM126A shRNA inhibits the growth of OSCC tumor *in vivo*. **(A)** Xenograft analysis of tumor growth *in vivo*. circFAM126A knockdown suppressed OSCC tumor growth. **(B, C)** Tumor weight and volume after transfection with circFAM126 knockdown. **(D)** The suppression of liver metastasis induced circFAM126 knockdown. **(E)** Expression of circFAM126 *in vivo* detected using qRT-PCR. **(F, G)** Expression of Ki67 in OSCC determined by immunohistochemistry. *P<0.05, **P<0.01.

Xenograft mouse models were established to determine the effects of circFAM126A shRNA on the growth of OSCC tumors *in vivo*. As shown in **Figures 4A–D**, knockdown of circFAM126A significantly decreased tumor size, weight, and volume, and liver metastasis; circFAM126A knockdown markedly decreased expression levels of circFAM126A and Ki67 (**Figures 4E** **–G**).

### miR-186 Is a Direct Target of circFAM126A

**Figure 5:**
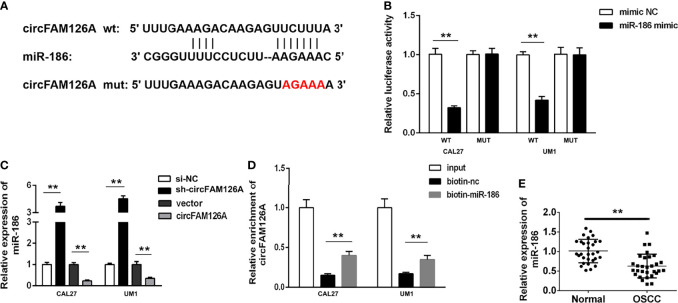
circFAM126A acts as a sponge of miR-186 in OSCC cells. **(A)** The binding sites predicted by Starbase3.0. **(B)** The binding sites verified by dual-luciferase reporter assay. **(C)** The expression of miR-186 detected using qRT-PCR. **(D)** The interaction between circFAM126A and miR-186 determined by RNA pull-down assay. **(E)** Expression of miR-186 in clinical samples determined by qRT-PCR. **P<0.01.

miR-186 was predicted to have binding sites for circFAM126A (**Figure 5A**). To confirm the targeting relationship between circFAM126A and miR-186, we performed luciferase activity reporter and RNA pull-down assays. As shown in **Figure 5B** (*P*<0.01), miR-186 mimics markedly decreased luciferase activity in wt-circFAM126A-transfected cells but had no significant effects on mut-circFAM126A-transfected cells. Furthermore, a circFAM126A probe and biotin miR-186-probe were used to perform the RNA pull-down assay. The results showed that the miR-186 probe could enrich circFAM126A (**Figure 5C**, *P*<0.01). Moreover, the expression of miR-186 in OSCC cells was significantly increased by knockdown of circFAM126A but significantly decreased by overexpression of circFAM126A (**Figure 5D**). As shown in **Figure 5E**, the expression of miR-186 was significantly decreased in OSCC tissues in comparison with adjacent normal tissues (*P*<0.01).

### RAB41 Is a Target of miR-186

**Figure 6:**
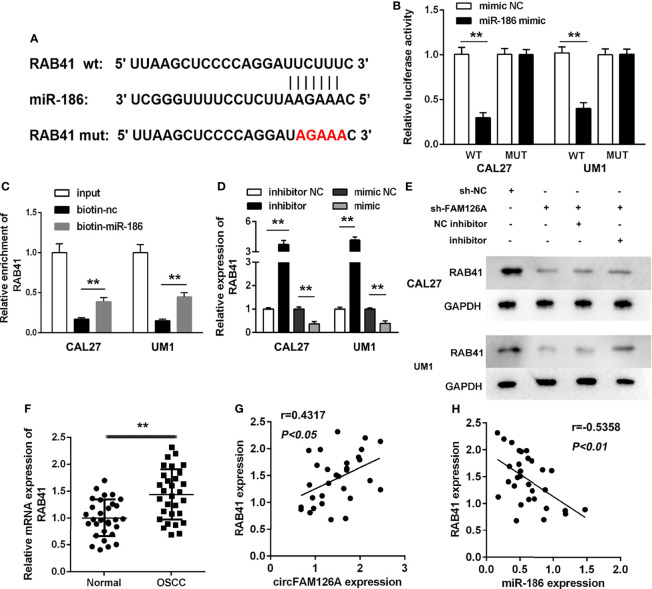
RAB41 is a target of miR-186 in OSCC cells. **(A)** The binding sites predicted by TargetScan7.2. **(B)** The binding sites verified by dual-luciferase reporter assay. **(C)** The interaction between RAB41 and miR-186 determined by RNA pull-down assay. **(D)** mRNA expression of RAB41 in OSCC cells detected using qRT-PCR. **(E)** Protein expression of RAB41 in OSCC cells measured using western blot. **(F)** Expression of RAB41 in OSCC clinical samples determined by qRT-PCR. **(G)** Correlation analysis of RAB41 and circFAM126A in clinical samples. **(H)** Correlation analysis of RAB41 and miR-186 in clinical samples. **P<0.01, ^##^ *P*<0.01.

miRNAs regulate biological progresses *via* binding to their targets. Online database TargetScan 7.2 was used to predict the targets of miR-186. **Figure 6A** showed the binding sites between miR-186 and RAB41 (**Figure 6A**). The luciferase assay showed that miR-186 mimics markedly decreased luciferase activity in wt-RAB41-transfected cells, whereas they had no significant effects on mut-RAB41-transfected cells (**Figure 6B**, *P*<0.01). The RNA pull-down assay further verified the interaction between miR-186 and RAB41 (**Figure 6C**, *P*<0.01). Overexpression of miR-186 significantly decreased the expression of RAB41 at both the mRNA and protein levels, whereas RAB41 expression was significantly upregulated by miR-186 inhibitors (**Figures 6D, E**, *P*<0.01). As shown in **Figure 6F**, the expression of RAB41 was significantly increased in OSCC tissues compared with adjacent normal tissues (*P*<0.01). The expression of RAB41 was positively correlated with circFAM126A expression and negatively correlated with miR-186 expression (**Figures 6G, H**, *P*<0.01).

### circFAM126A Interacts With FUS to Promote mRNA Stability of RAB41

**Figure 7:**
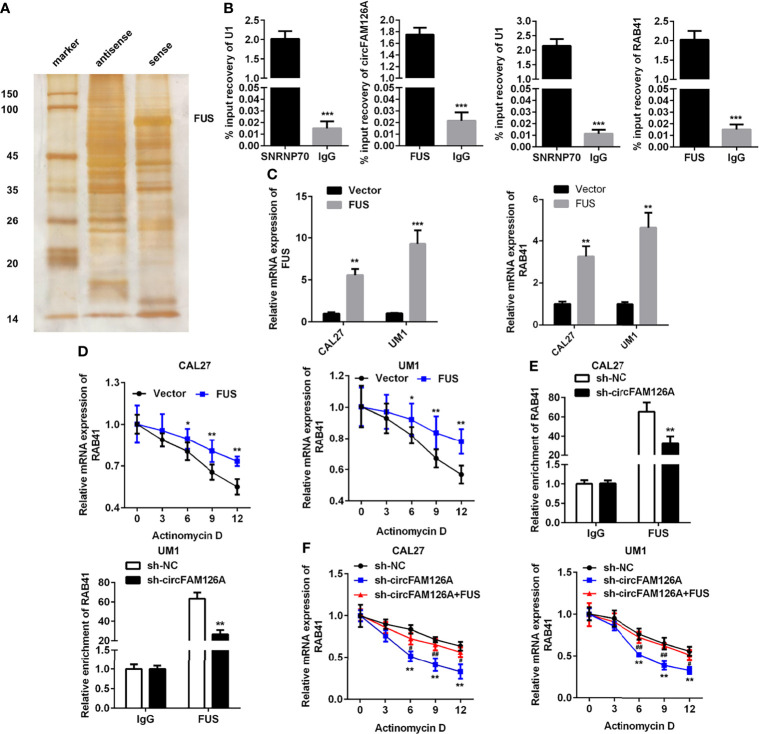
circFAM126A binds to FUS to promote the mRNA stability of RAB41. **(A)** The potential proteins interacting with circFAM126A detected using RNA pull-down. **(B)** Interaction between FUS and circFAM126A or RAB41 confirmed by RIP assay. **(C)** mRNA expression of FUS and RAB41 detected by qRT-PCR. **(D)** mRNA stability of RAB41 determined using qRT-PCR. **(E)** Interaction between FUS and circFAM126A or RAB41 verified by RIP assay. **(F)** mRNA stability of RAB41 determined using qRT-PCR. *P<0.05, **P<0.01 and ***P<0.001.

circRNAs regulate gene expression *via* binding to miRNAs or RNA-binding proteins (RBPs). RNA pull-down and mass spectrometry analyses showed that circFAM126A could bind to FUS (**Figure 7A**, *P*<0.01). The RIP assay further verified the interaction between FUS and circFAM126A and RAB41 (**Figure 7B**, *P*<0.001). The expression of FUS was significantly increased after transfection with FUS, indicating that cells had been successfully transfected (**Figure 7C**, *P*<0.01, *P*<0.001). Moreover, overexpression of FUS significantly increased its mRNA stability (**Figure 7D**, *P*<0.05, *P*<0.01), and circFAM126A modulated the interaction between FUS and RAB41 (**Figure 7E**, *P*<0.01). The decrease in mRNA stability induced by circFAM126A knockdown was reversed by overexpression of FUS (**Figure 7F**, *P*<0.01).

### circFAM126A Regulates the Proliferation and EMT of OSCC Cells *via* Targeting the miR-186/RAB41 Axis

**Figure 8:**
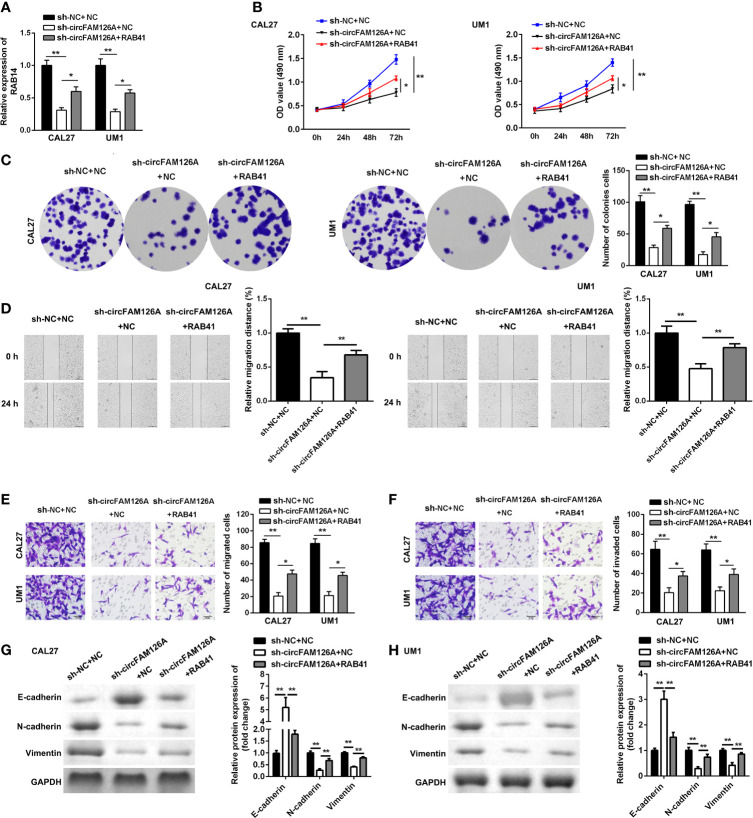
Overexpression of RAB41 reverses the anti-tumor effects of sh-circFAM126A. **(A)** Expression of RAB41 in OSCC cells detected using qRT-PCR. **(B, C)** Viability and proliferation ability of OSCC cells detected by MTT and colony formation assay. **(D)**. Migration determined using wound healing assay. **(E, F)** Migration and invasion ability of OSCC cells measured using transwell assay. **(G, H)** Protein expressed determined using western blot. *P < 0.05, **P < 0.01.

As shown in **Figure 8A**, RAB41 overexpression plasmids significantly increased the expression of RAB41 compared with sh-circFAM126A (*P*<0.01). Moreover, compared with circFAM126A knockdown, overexpression of RAB41 significantly promoted the proliferation (**Figures 8B, C**, *P*<0.01), migration (**Figures 8D, E**, *P*<0.01), and invasion (**Figure 8F**, *P*<0.01) of OSCC cells *in vitro*. Additionally, upregulated RAB41 antagonized the effects of circFAM126A knockdown on the protein expression of Snail, Vimentin, E-cadherin, and N-cadherin (**Figures 8G, H**, *P*<0.01).

## Discussion

Dysregulated circRNAs play a crucial role in the development of OSCC. circRNAs may function as anti-tumor genes or oncogenes in OSCC. In this study, we found that circFAM126A was upregulated in OSCC. Moreover, SP1-induced upregulation of circFAM126A promoted the proliferation and EMT of OSCC cells by regulating the miR-186/FUS/RAB41 axis *in vitro*. However, knockdown of circFAM126A suppressed the aggressiveness and metastasis of OSCC *in vitro* and *in vivo*. This is the first study to investigate the mechanism, expression, regulation, and clinical implications of circFAM126A in OSCC.

circRNAs are a type of non-coding RNA. Owing to their stability, diversity, high expression, and high sequence conservation, and other biological characteristics, circRNAs have potential as diagnostic markers and therapeutic targets in tumorigenesis and progression. Many studies have reported abnormal expression of circRNAs in different types of tumors. For example, Zhang et al. (24) found evidence that circ-100876 is downregulated in colorectal cancer tissues, and that low expression of circ-100876 predicts poor prognosis and increases the risk of relapse of colorectal cancer patients. Yu et al. (25) found that the expression of circRNA SMARCA5 is significantly downregulated in liver cancer tissues and is associated with early tumor stage and poor prognosis of liver cancer patients. Moreover, circRNAs play vital roles in many physiological and pathological processes, including cell cycle progression, autophagy, proliferation, invasion, metastasis, and carcinogenesis (26, 27). In the present study, circFAM126A was found to be upregulated in OSCC. Moreover, knockdown of circFAM126A markedly inhibited OSCC cell proliferation and EMT of OSCC cells, manifested by the upregulation of epithelial marker (E-cadherin) and downregulation of mesenchymal markers (N-cadherin and Vimentin) (17–21). Additionally, circFAM126A knockdown inhibited tumor growth and metastasis of OSCC *in vivo*. Therefore, the above data suggest that circFAM126A may have an oncogenic role in the progression of OSCC.

Previous reports demonstrate that transcription factors participate in the progression of cancer (28–30). Dysregulated transcription factors promote tumorigenesis *via* transcriptionally regulating non-coding RNAs, such as long coding RNAs and circRNAs (29). We further investigate the upstream of circFAM126A. SP1 functions as an oncogene in various cancer, including OSCC (31–33). In this study, SP1 was overexpressed in OSCC tissues and cells. Moreover, SP1 transcriptionally upregulated circFAM126A, which further contributed the tumor growth and metastasis of OSCC.

Accumulating evidence demonstrates that miRNAs have essential roles in the regulation of tumor progression (34–36), and circRNAs might exert their functions through targeting miRNAs. miR-186 has been suggested to function as a tumor suppressor in the progression of OSCC (37). In this study, miR-186 was predicted and proved to be a target of circFAM126A. In addition, circFAM126A interacted with miR-186 to regulate the proliferation and EMT of OSCC cells. Therefore, circFAM126A may exert its carcinogenic function *via* targeting miR-186.

circRNAs, which lack the ability to encode proteins, function as ceRNAs to regulate gene expression *via* binding to miRNAs or RBPs (38). The circRNA/(miRNA/RBP)/mRNA axis may intensively participate in the progression of cancers including OSCC (39, 40). In this study, circFAM126A modulated the expression of RAB41 *via* sponging miR-186 and promoted its mRNA stability *via* interacting with RNA binding protein FUS. RNA pull-down assay and mass spectrometry analysis showed that circFAM126A could bind to FUS to increase the mRNA stability of RAB41. circFAM126A increased the expression of RAB41 *via* sponging miR-186. These results further elucidated the underlying mechanism, in which circFAM126A increased the mRNA expression of RAB41. RAB41 is a member of RAB family, which frequently acts as oncogenes in various cancer (41). RAB41 plays an essential role in membrane trafficking, high expression of which is associated with poor clinical results of lung adenocarcinoma (42). However, the roles of RAB41 in OSCC have not been fully elucidated. In this study, RAB41 was found to be overexpressed in OSCC. The expression of circFAM126A was positively correlated with RAB41. Overexpression of RAB41 alleviated the effects of circFAM126A knockdown and promoted an aggressive phenotype of OSCC cells. Taken together, these results suggest that circFAM126A regulates the growth and metastasis of OSCC cells *via* modulation of the miR-186/FUS/RAB41 axis.

## Conclusion

Taken together, circFAM126A played vital roles in the progression of OSCC. SP1-mediated upregulation of circFAM126A promoted the growth and metastasis of OSCC cells *via* the miR-186/FUS/RAB41 axis. These results could indicate a new target for the treatment of OSCC.

## Data Availability Statement

The original contributions presented in the study are included in the article/supplementary material. Further inquiries can be directed to the corresponding author.

## Ethics Statement

The studies involving human participants were reviewed and approved by Affiliated Stomatological Hospital of Nanchang University. The patients/participants provided their written informed consent to participate in this study.

## Author Contributions

JW was responsible for the organization and coordination of the trial. SO was the chief investigator and responsible for the data analysis. SZ, XZ, MC, XF, YC, and LL developed the trial design. All authors contributed to the writing of the final manuscript.

## Funding

This study was supported by National Nature Foundation (82160194 and 81960492), Jiangxi Natural Science Foundation (20192BAB205054 and 20181ACB20022), General Projects of Jiangxi Province (20192BBG70023).

## Conflict of Interest

The authors declare that the research was conducted in the absence of any commercial or financial relationships that could be construed as a potential conflict of interest.

## Publisher’s Note

All claims expressed in this article are solely those of the authors and do not necessarily represent those of their affiliated organizations, or those of the publisher, the editors and the reviewers. Any product that may be evaluated in this article, or claim that may be made by its manufacturer, is not guaranteed or endorsed by the publisher.
